# Fetuin-A levels in association with calcific aortic valve disease: A meta-analysis^☆^

**DOI:** 10.1016/j.athplu.2023.09.004

**Published:** 2023-09-26

**Authors:** Muhammad Omar Larik

**Affiliations:** Department of Medicine, Dow International Medical College, Karachi, Pakistan

**Keywords:** Fetuin-A, Calcific aortic valve disease, Aortic stenosis, Aortic sclerosis

## Abstract

**Background and aims:**

Calcific aortic valve disease (CAVD) is a common valvular disease, prevalent particularly within the older age groups. The potential use of biomarkers in diagnosing and assessing the severity of CAVD, in supplementation with imaging techniques, has recently gained momentum within the field of cardiovascular medicine. Therefore, a meta-analysis was performed that assessed the association between the fetuin-A levels, and the presence of CAVD.

**Methods:**

PubMed and Cochrane were searched from inception to April 2023. Risk of bias was assessed using the Newcastle-Ottawa scale for cohort studies.

**Results:**

This analysis includes a total of 3,280 patients with CAVD, and 7,505 patients as control, resulting in the pooling of 10,785 patients in this meta-analysis. It was observed that the circulating levels of fetuin-A were significantly lowered in patients with CAVD (SMD: -0.20; 95% CI: -0.39, -0.02; P = 0.03). Moreover, the analysis revealed that fetuin-A levels had no significant association with CAVD in patients suffering from kidney disease (SMD: 0.20; 95% CI: -0.46, 0.85; P = 0.56).

**Conclusion:**

While initial results demonstrate the potential effectiveness, further research is essential in order to arrive at a robust conclusion regarding the use of fetuin-A as a diagnostic biomarker for calcific aortic valve disease.

## Highlights

• Calcific aortic valve disease (CAVD) is a common valvular disease.
• Fetuin A was significantly decreased in patients with CAVD.
• Fetuin A as a diagnostic biomarker may be ineffective in kidney disease patients.
• Further research is needed in order to arrive at a robust conclusion.

## 1 Introduction

In developed countries, aortic stenosis is the most common valvular disorder associated with calcification of the valve. This is an alarmingly common disease, with prevalence of up to 1% in children, 25% in older adults aged >65 years, and up to 50% in the elderly aged >85 years [1]. The pathophysiology of calcific aortic valve disease (CAVD) usually pertains to the complex processes of progressive mechanical and oxidative stress, endothelial dysfunction, inflammatory processes, and calcification [1,2]. Aortic sclerosis is the earliest manifestation of calcific aortic valve disorder, which occurs due to the increasing calcification of the cusps. Recently, the use of biomarkers in assessing the calcification of aortic valves has gained momentum in the field of research. Although the gold standard for screening and diagnosis includes imaging techniques such as echocardiography, the utilization of routine testing can be conducted through circulating biomarker concentrations, allowing the possibility for quantitative assessment of the degree of aortic calcification and CAVD. Fetuin-A is a glycoprotein that is synthesized in the liver, and functions as a putative target against calcific valve disease, and a potent inhibitor of tissue calcification [3,4]. In recent research, fetuin-A has shown an acute phase response to injury and inflammation, and may serve as a protective agent to systemic inflammation, with associations between cardiovascular disease (coronary artery disease, valvular stenosis, and calcification) also observed [5]. Therefore, in this meta-analysis, the association between circulating levels of fetuin-A and CAVD was assessed via the pooling of relevant studies.

## 2 Methods

Databases, including PubMed and Cochrane, were independently searched for relevant studies, from inception to April 2023. The following keywords were used: “fetuin-A″, “alpha-2-HS-glycoprotein”, “AHSG”, “calcific aortic valve disorder”, “aortic valve stenosis”, “aortic valve sclerosis”, and “aortic valve calcification”. Any original studies comparing the serum values of fetuin-A with a control were included. Systematic reviews, narrative reviews, case reports, and letters were excluded through the screening process. The primary outcome of this meta-analysis was the association of circulating fetuin-A levels with the presence of CAVD. Review Manager (Version 5.4: Cochrane Collaboration, 2020) was used for statistical analysis, with the pooling of standard mean difference values and the random-effects model employed. A subgroup analysis was performed due to certain studies reporting patient data with various forms of kidney disease, or patients undergoing hemodialysis due to kidney disease.

The initial search revealed 14 studies that were potentially relevant. After a comprehensive full-text screening, nine studies were selected [2,3,[6], [7], [8], [9], [10], [11], [12]], including 3,280 patients with CAVD and 7,505 patients as controls. The Newcastle-Ottawa scale for bias assessment was utilized to determine the quality of the included studies. The baseline characteristics of the included patient population and the risk of bias ratings are summarized in Table 1.

**Table 1:** Baseline characteristics of included studies.

Study	CAVD, n	Control, n	Mean Age, y (SD)		Male, n		Hypertension (%)		Diabetes (%)		Smoking (%)		Creatinine, mg/dL (SD)		Risk of Bias
			CAVD	Control	CAVD	Control	CAVD	Control	CAVD	Control	CAVD	Control	CAVD	Control	
Sainger et al. [2]	54	59	76.1 ± 1.1	62.9 ± 2.2	30	29	74%	73%	28%	20%	41%	37%	–	–	★★★★★★
Ossareh et al. [3]	92	30	63 ± 15	47 ± 15	55	19	15%	3%	51%	30%	–	–	8.8 ± 2.8	10.8 ± 3	★★★★★
Ferrari et al. [6]	33	11	75.9 ± 7.2	55.4 ± 24.2	15	8	82%	64%	36%	27%	33%	9%	–	–	★★★★★★★
Kaden et al. [7]	31	28	72 ± 7	69 ± 5	14	15	74%	79%	26%	21%	42%	43%	1.2 ± 0.3	1.1 ± 0.2	★★★★★
Adamczyk et al. [8]	68	20	68.6 ± 7.7	66.1 ± 7.7	39	12	85%	85%	24%	30%	38%	30%	–	–	★★★★★★
Bortnick et al. [9]	1,517	1,850	72 ± 5	71 ± 4	586	665	–	–	13%	12%	52%	53%	–	–	★★★★★
Bortnick et al. [10]	913	5,899	70 ± 8	61 ± 10	549	2,663	55%	34%	20%	12%	57%	49%	–	–	★★★★★★
Kocyigit et al. [11]	51	14	41.2 ± 12.3	33.1 ± 11.7	33	12	–	–	–	–	–	–	1 ± 0.5	1.3 ± 1.0	★★★★★★
Koca et al. [12]	11	45	39.1 ± 11.2	36.9 ± 10.9	3	24	69%	69%	9%	20%	0%	13%	1.43 ± 0.64	1.42 ± 0.56	★★★★★★

CAVD, Calcific Aortic Valve Disease; n, Number of Patients; y, Years; SD, Standard Deviation.

## 3 Discussion

**Fig. 1:**
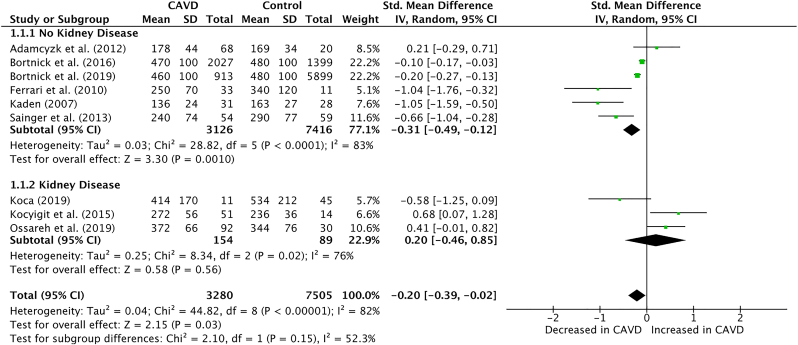
Forest plot of association of Fetuin-A levels with Calcific Aortic Valve Disease.

This meta-analysis revealed that the circulating levels of fetuin-A were significantly lowered in patients with aortic valve calcifications or stenosis, compared to the control group with no calcifications (SMD: -0.20; 95% CI: -0.39, -0.02; P = 0.03; I^2^ = 82%; Fig. 1). Additionally, a subgroup analysis was performed in studies reporting fetuin-A levels in patients with kidney disease. This analysis revealed that fetuin-A had no significant association with CAVD in patients suffering from kidney disease (SMD: 0.20; 95% CI: -0.46, 0.85; P = 0.56; I^2^ = 76%; Fig. 1). However, there were no significant subgroup differences observed between the two cohorts (P = 0.15). CAVD is a multifactorial disorder with complex pathophysiology and biochemistry [13]. There have been other biomarkers, such as plasma osteopontin and parathyroid hormone, which have shown an increased serum levels in patients with CAVD [2,6]. This paves the road to generate a range of serum biomarkers, in conjunction with fetuin-A, that may be used to identify valvular calcification, the staging of valvular calcification, and progression in patients with aortic stenosis or sclerosis, which could supplement the current standard of cardiac imaging. Moreover, it was noted that the subgroup suffering from kidney diseases did not demonstrate any significant differences when compared to the control. This is an extremely consequential finding, as the risk of valvular calcification is four to five times greater in patients with end-stage kidney disease as compared to the general population [3]. This may be explained by the lack of change in fetuin-A levels in chronic kidney disease patients, who typically observe a rise in fetuin mineral complex (FMC) [3]. However, there were no statistically significant subgroup differences between the two cohorts, illustrating the need for further comprehensive research regarding the impact of FMC on CAVD in patients with kidney disease is encouraged, in order to arrive at a valid conclusion. This meta-analysis includes several limitations. Firstly, the unadjusted data may account for some inconsistencies, as populations of certain studies were subject to a variation of comorbidities when compared to other studies, such as the presence of hypertension, diabetes mellitus, or smoking history. Secondly, this meta-analysis was exclusively performed using observational studies, potentially accounting for an increased level of heterogeneity due to residual bias and the presence of confounding factors in studies of observational nature. Therefore, future high-powered randomized studies featuring patient-level adjustments is strongly indicated, in order to further consolidate our findings.

## 4 Conclusion

In conclusion, it was observed that fetuin-A was significantly decreased in patients with known calcific aortic valve disease. However, there was no association between fetuin-A and calcific aortic valve disease in patients with kidney disease. Further research is essential in order to arrive at a robust conclusion regarding the use of fetuin-A as a diagnostic biomarker for calcific aortic valve disease.

## Statements and declarations

The authors declare that they have no competing or conflicting interests.

The authors declare that they received no funds, grants, or support.

IRB approval was not required for this paper.

## Declaration of competing interest

The authors declare that they have no known competing financial interests or personal relationships that could have appeared to influence the work reported in this paper.
